# Inherited *GATA3* variant associated with positive minimal residual disease in childhood B‐cell acute lymphoblastic leukemia via asparaginase resistance

**DOI:** 10.1002/ctm2.507

**Published:** 2021-08-23

**Authors:** Chunjie Li, Wenyi Liang, Yingyi He, Xinying Zhao, Jiabi Qian, Ziping Li, Chuang Jiang, Qingqing Zheng, Xiangmeng Fu, Weina Zhang, Haiyan Liu, Xin Sun, Maoxiang Qian, Hui Zhang

**Affiliations:** ^1^ Department of Hematology/Oncology, Affiliated Guangzhou Women and Children's Medical Center, Zhongshan School of Medicine Sun Yat‐sen University Guangzhou China; ^2^ Institute of Pediatrics, Affiliated Guangzhou Women and Children's Medical Center, Zhongshan School of Medicine Sun Yat‐sen University Guangzhou China; ^3^ Department of Hematology/Oncology, Guangzhou Women and Children's Medical Center Guangzhou Medical University Guangzhou China; ^4^ Shanghai Children's Medical Center, School of Medicine Shanghai Jiaotong University Shanghai China; ^5^ Institute of Pediatrics and Department of Hematology and Oncology, Children's Hospital of Fudan University, National Children's Medical Center, the Shanghai Key Laboratory of Medical Epigenetics, International Co‐laboratory of Medical Epigenetics and Metabolism (Ministry of Science and Technology), Institutes of Biomedical Sciences Fudan University Shanghai China; ^6^ National Children's Medical Center, Department of Hematology/Oncology, Key Laboratory of Pediatric Hematology and Oncology of China Ministry of Health, Shanghai Children's Medical Center, School of Medicine Shanghai Jiao Tong University Shanghai China

Dear Editor,

Genome‐wide association studies have identified that germline single nucleotide polymorphisms (SNPs) in *GATA3* significantly influence the treatment outcomes of childhood acute lymphoblastic leukemia (ALL).^1^, ^2^, ^3^ However, the role of inherited *GATA3* variants in Han Chinese patients with B‐cell ALL (B‐ALL) and the molecular mechanisms by which these variants are linked to poor prognosis are largely unknown.

We genotyped *GATA3* SNPs rs3824662 and rs3781093 in 308 children with B‐ALL enrolled in the CCCG‐ALL‐2015 study to evaluate their association with ALL treatment outcomes in the Han Chinese population (Figure 1A, Table S1). Using an additive logistic regression model, we found that *GATA3* rs3824662 A allele and rs3781093 C allele were significantly associated with minimal residual disease (MRD) positivity on day 46 (*p *= 0.039, odds ratio [OR] = 1.54 [95% confidence interval: 1.01–2.36], and *p *= 0.036, OR = 1.55 [1.03–2.39] in dichotomous analysis, respectively; *p *= 0.02 and *p *= 0.018 in ordinal analysis, respectively) (Figure 1B,C, Figures S1–S3). The A allele of rs3824662 and C allele of rs3781093 were both >1.5‐fold increased odds ratio for risk of MRD positivity compared with their reference alleles (Figure 1B,C). To validate the association of *GATA3* SNPs with MRD, we genotyped rs3824662 and rs3781093 in 122 children from another B‐ALL cohort enrolled in the GD‐2008‐ALL study (Table S2). In this replication analysis, risk alleles of both SNPs were consistently over‐represented in MRD positive patients (day 33) compared to that in MRD negative patients: rs3824662 (*p* = 0.015, OR = 2.06 [1.18–3.59]) and rs3781093 (*p* = 0.022, OR = 1.95 [1.11–3.43]) in dichotomous analysis, and *p *= 0.050 and *p *= 0.078 in ordinal analysis, respectively (Figure 1B,C, Table S3).

**FIGURE 1:**
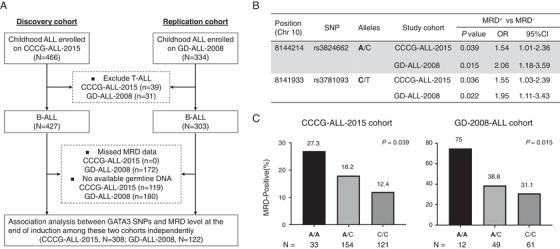
*GATA3* rs3824662 was associated with positive MRD in pediatric Han Chinese B‐ALL patients. (A) Flowchart of the candidate gene association study. Sanger sequencing of *GATA3* rs3824662 and rs3781093 in the discovery CCCG‐ALL‐2015 cohort (466 patients) and validation GD‐2008‐ALL cohort (334 patients) was performed, followed by analysis of the association between genotype and minimal residual disease (MRD) status. (B) Association of the genotype of *GATA3* SNPs rs3824662 and rs3781093 with end‐of‐induction MRD; the threshold was set as <0.01% with *p* values estimated through logistic regression analysis. (C) The frequency of A allele of rs3824662 in MRD‐positive state among CCCG‐ALL‐2015 and GD ‐2008‐ALL cohorts

To investigate the biological function of the germline *GATA3* variant, we examined the chromatin state of this genomic region across different hematopoietic cell types by ChromHMM.^4^ Across 11 hematopoietic cells, we interestingly found that rs3824662 was resided inside regions with weak enhancer activity in hematopoietic tissues (Figure 2A), suggesting the cis‐transcriptional regulation role. To further strengthen our findings, we next retrieved GM12788 ChIA‐PET and epigenetic data and identified that rs38246622 located in RNA Pol II peak anchor regions with the enrichment of histone marks H3K27ac and H3K4me1, while lacking H3K27me3 signal (Figure 2B), consolidating the enhancer role of rs3824662. To confirm the risk allele on enhancer function, we evaluated the impact of *GATA3* variants on its transcription activity using a luciferase reporter assay. Surprisingly, the rs3824662 A risk allele significantly increased the enhancer activity by approximately threefold compared to the nonrisk allele in GM18900, Nalm6, and Reh cells, while the rs3781093 C risk allele did not affect *GATA3* transcription (Figure 2C, Figure S4). To further confirm the enhancer activity of rs3824662 A allele on *GATA3* transcription, we converted the original wild‐type C allele to A allele at rs3824662 in the lymphoblastoid cell line GM18900 using CRISPR/Cas9 system. Engineered cells with A/A or A/C genotype exhibited significantly higher *GATA3* expression (approximately threefold) compared with the parental cells with C/C genotype, independent of the allele frequency (Figure 2D, Figure S5). Taken together, these results provided a clue to the link between the biological function of rs3824662 and its association with MRD.

**FIGURE 2:**
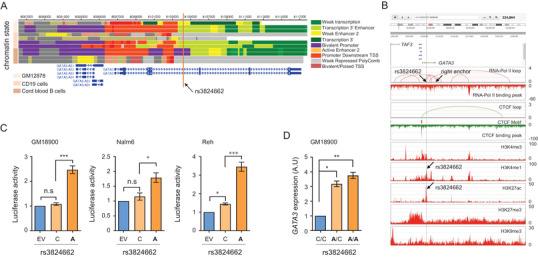
Location and effect of rs3824662 on *GATA3* expression. (A) Chromatin state annotations from the Roadmap Epigenomics Project. The chromatin states were plotted across the *GATA3* genomic region of human B lymphocytes. These epigenomic data suggest that rs3824662 is located inside a hematopoietic cell‐specific enhancer element (orange line). (B) Genomic browser screenshot defined the enhancer activity of *GATA3* rs3824662 with RNA‐Pol II loop, CTCF loop, and ChIP‐seq signals for histone marks shown as legend in GM12878 cells. (C) Luciferase reporter assay comparing the enhancer activities of the fragments containing either rs3824662 risk A allele or wildtype C allele in GM18900 cells, an immortalized B lymphoblastoid cell lines with wildtype C allele, and Nalm6 and Reh B‐ALL cell lines. (D) Cis effects of rs3824662 A allele on *GATA3* expression. The expression of each gene in wild‐type (C/C) and engineered GM18900 cells (A/A and A/C) was quantified using qRT‐PCR. All experiments were performed in triplicate and repeated three independent times. Bars represent the mean values; the error bars represent the SD from triplicate. ns, no significance; *p < 0.05; **p < 0.01; ***p < 0.001 (Student's *t*‐test)

We speculated that active *GATA3* expression might lead to drug resistance, a major contributor to MRD.^5^ To test this hypothesis, we retrieved a series of expression profiling array datasets from the NCBI GEO database and investigated a correlation between *GATA3* expression and the drug sensitivity of primary B‐ALL cells.^6^ High levels of *GATA3* expression were significantly correlated with l‐asparaginase (l‐Asp, *p *< 0.0001) (Figure 3A, Figures S6 and S7). To confirm the correlations, we tested the drug response in established B‐ALL cell lines (697 and SUP‐B15) with ectopic overexpression and knockdown of *GATA3* using MTT assay. As shown in Figure 3B, l‐Asp resistance induced by *GATA3* overexpression was completely rescued by *GATA3* knockdown in *GATA3* overexpression cells (Figure 3B). The association of *GATA3* expression with l‐Asp resistance was also confirmed in nine primary B‐ALL samples (Figure 3C,D).

**FIGURE 3:**
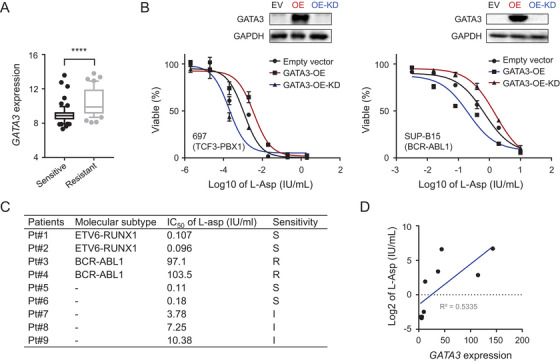
Correlation between *GATA3* expression and l‐Asp resistance in B‐ALL cells. (A) High *GATA3* expression was significantly correlated with l‐Asp resistance. Gene expression was compared between l‐Asp‐sensitive and l‐Asp‐resistant B‐ALL cells from GSE653 and GSE654 datasets. Each box plot shows the distribution of log2 values of *GATA3* transcription from the 10th to the 90th percentile. The line inside each box plot represents the median; ****p < 0.0001 (Unpaired *t*‐test). (B) l‐Asp response of B‐ALL cell lines (left panel, 697 cells [TCF3‐PBX1 fusion]; right panel, SUP‐B15 cells [BCR‐ABL1 fusion]) with ectopic *GATA3* expression (red curve) and *GATA3* knockdown in prior *GATA3* ectopic expression (blue curve). Drug sensitivity was detected using MTT assay. (C) l‐Asp sensitivity and *GATA3* transcription were tested in nine primary B‐ALL cells. Primary leukemia cell samples (*n* = 9) isolated from the patient's peripheral blood samples were treated with different concentrations of l‐Asp and IC_50_ values were calculated after plotting l‐Asp dose‐dependent survival of leukemic cells measured by MTT cell viability assay. l‐Asp sensitivity was classified into three categories: “susceptible (S),” “intermediate (I),” and “resistant (R)” according to the IC_50_ value. (D) l‐Asp sensitivity was plotted against *GATA3* transcription to determine their association. *X* and *Y* axes represent relative *GATA3* expression level and IC_50_ value to l‐asp (shown as log2), respectively

Several potential l‐Asp resistance mechanisms have been confirmed within different contexts, but none of them related to *GATA3* expression (Figures S8–S10). Takahashi et al. identified that autophagy was essential for cell survival under l‐Asp‐induced stress in ALL cells.^7^ To test the role of autophagy in *GATA3*‐induced l‐Asp resistance, we next evaluated autophagy flux in 697 cells. By western blotting, LC3B‐II levels were observed to be increased with *GATA3* overexpression, and this increase was more obvious with l‐Asp treatment (Figure 4A). To further determine how the active expression of *GATA3* induces autophagy activation, we evaluated the expression of two key autophagy‐related genes (*BECN1* and *ATG5*). As shown in Figure 4B, overexpression of *GATA3* induced upregulation of these two genes at mRNA levels. Furthermore, the promoter activity of *BECN1* and *ATG5* was increased upon the overexpression of *GATA3* in HEK293T (*p *= 0.0098 and 0.0114, respectively; Figure 4C), indicating that *GATA3* can regulate the transcription of key autophagy‐related genes. Finally, we inhibited autophagosome turnover in 697 cells with chloroquine diphosphate salt (CQ) and found that *GATA3*‐induced l‐Asp resistance was almost completely rescued (Figure 4D), suggesting the potential mechanism of *GATA3* mediated l‐Asp resistance via activation of autophagy.

**FIGURE 4:**
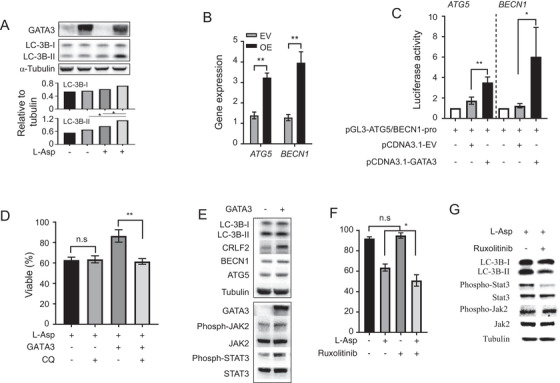
*GATA3* induced l‐Asp resistance via JAK2‐STAT3‐mediated autophagy activation. (A) Immunoblotting was performed to determine the effects of l‐Asp and *GATA3* on autophagy. Fold change of LC3B‐I and LC3B‐II level (normalized to α‐tubulin) relative to that of untreated cells is indicated in the graph in the lower panel. (B) Expression of *ATG5* and *BECN1* in 697 cells with or without *GATA3* ectopic expression was quantified using qRT‐PCR. (C) Luciferase reporter assay was used to determine the transactivation effects of *GATA3* on autophagy‐related genes *ATG5* and *BECN1*. Highly conserved sequence from *BECN1* or *ATG5* promoter region was cloned into luciferase reporter constructs. Overexpression of *GATA3* dramatically increased luciferase activity compared with the corresponding empty vector. (D) CQ completely rescued the l‐Asp resistance induced by *GATA3* overexpression. (E) Immunoblots were used to determine the effects of *GATA3* on autophagy and JAK2‐STAT3 signaling pathway. (F) Ruxolitinib treatment could partially rescue the l‐Asp resistance in 697 cells with *GATA3* ectopic expression. Note that 697 cells were treated with ruxolitinib (1 μM) and/or l‐Asp (2 mIU/ml) for 48 h. Drug sensitivity was detected through MTT cell viability assay. (G) Immunoblots were used to determine the effects of ruxolitinib on autophagy and JAK2‐STAT3 signaling pathway. B‐ALL cells were treated with ruxolitinib (1 μM) and/or l‐Asp (2 mIU/mL) for 6 h. All experiments were performed in triplicate and repeated three independent times. Bars represent the mean values; the error bars represent the SD from triplicate. ns, no significance; *p < 0.05; **p < 0.01 (Student's *t*‐test)

To gain more insights into the mechanism of *GATA3* mediated l‐Asp resistance, we determined whether *GATA3* can regulate the JAK‐STAT signaling pathway.^8^ As shown in Figure 4E, *GATA3* overexpression resulted in increased expression of CRLF2 and phosphorylation of JAK2 and STAT3. Intriguingly, inhibition of JAK2‐STAT3 signaling by ruxolitinib suppressed autophagy activation, which in turn sensitized B‐ALL cells to l‐Asp treatment (Figure 4F,G), indicating another layer of regulation of autophagy by *GATA3* via posttranslation regulation of JAK2‐STAT3 signaling in B‐ALL cells.

In this work, we first validated that *GATA3* rs3824662 was associated with the risk of MRD after induction treatment in Han Chinese children with ALL. Mechanistic studies showed that rs3824662 *cis*‐promoted *GATA3* expression, which in turn induced l‐Asp resistance via CRLF2‐JAK2‐STAT3‐related autophagy activation. These findings will be of value in upfront risk stratification of childhood B‐ALL and enrich our understanding of the role of *GATA3* in ALL pathogenesis and prognosis.

## CONFLICT OF INTEREST

All the authors have no conflict of interest to declare.

## AUTHORS CONTRIBUTION

The study was conceived by Hui Zhang, designed by Chunjie Li, Maoxiang Qian, Hui Zhang, and supervised by Maoxiang Qian and Hui Zhang. Hui Zhang and Chunjie Li performed the CRISPR/Cas‐9 and ectopic *GATA3* expression experiments. Chunjie Li, Xinying Zhao, Jiabi Qian, and Ziping Li performed genotyping in these two cohorts. Hui Zhang and Chunjie Li performed drug tests in primary ALL samples and ALL cell lines. Chunjie Li and Xinying Zhao performed the molecular experiments. Yingyi He enrolled the patients and performed the clinical data analysis. Data analysis was conducted by Chunjie Li, Chuang Jiang, and Hui Zhang, statistical analyses by Chunjie Li and Hui Zhang; data interpretation by Chunjie Li, Wenyi Liang, Yingyi He, Xinying Zhao, Jiabi Qian, Ziping Li, Chuang Jiang, Qingqing Zheng, Xiangmeng Fu, Weina Zhang, Haiyan Liu, Xin Sun, Maoxiang Qian, and Hui Zhang. Chunjie Li, Maoxiang Qian, and Hui Zhang wrote the manuscript. All authors approved the final version for publication.

## DATA AVAILABILITY STATEMENT

The data that support the findings of this study are available from the corresponding authors upon reasonable request.

## ETHICS APPROVAL AND CONSENT TO PARTICIPATE

This study was approved by the institutional ethics committee of Guangzhou Women and Children's Medical Center (IRB No. 2018022205, 2017102307, 2015020936), registered in the Chinese Clinical Trial Registry (ChiCTR‐POC‐17013315), and performed in accordance with the Declaration of Helsinki. Informed consent was obtained from patients or their guardians.

## Supporting information

Supporting Information

Supporting Information

Supporting Information
