# Supplementary material for: Inherited GATA3 variant associated with positive minimal residual disease in childhood B‐cell acute lymphoblastic leukemia via asparaginase resistance
Source: Clin Transl Med. 2021 Aug 23;11(8):e507. doi: 10.1002/ctm2.507 (PMC8382977; doi:10.1002/ctm2.507)
Supplement: Supplementary file 1 — Supporting Information [file CTM2-11-e507-s002.docx]

**Supplementary Information**

**Materials and Methods**

**Patients**

The discovery cohort consisted of 308 patients prospectively enrolled in the Chinese Children’s Cancer Group (CCCG)-ALL-2015 clinical trial. The replication cohort consisted of 122 children with ALL treated with the Guangdong (GD)-2008-ALL protocol. Details of the enrollment criteria and study design have been described previously^1,2^. All the investigated pediatric ALL patients were children with newly diagnosed B-precursor ALL treated at the Guangzhou Women and Children’s Medical Center, Guangzhou, China (**Supplementary Table 1, 2**).

**Ethical statement**

This study was approved by the institutional ethics committee of Guangzhou Women and Children’s Medical Center (IRB No. 2018022205, 2017102307, 2015020936), registered in the Chinese Clinical Trial Registry (ChiCTR-POC-17013315), and performed in accordance with the Declaration of Helsinki. Informed consent was obtained from patients or their guardians.

**MRD assessment**

MRD data were assessed using standardized flow cytometry. An MRD level of 0.01% (1 leukemic cell/10,000 normal cells) or higher was defined as MRD positivity in our study. MRD status was categorized as negative (<0.01%), positive (>=0.01%, but <1%), and high positive (>=1%). Patients whose specimens were not available or whose MRD data were missing at the end of induction therapy were excluded from this study. In total, 308 patients from CCCG-ALL-2015 and 122 patients from GD-2008-ALL were included for analysis.

**Association and validation analyses**

The association between MRD status and genotypes at *GATA3* rs3824662 and rs3781093 was tested. First, MRD was treated as a dichotomous variable (that is, MRD-positive ≥0.01% leukemic cells or MRD-negative <0.01% leukemic cells) and modeled with logistic regression using SNP as a predictor. Secondly, MRD was treated as an ordinal variable (<0.01%, ≥0.01%–<0.1%, ≥0.1%–<1%, ≥1%), and its association with SNP was evaluated using a Spearman-type correlation. In both discovery and replication analyses, the significance threshold was set at *P* <0.05.

**Materials**

All cells were cultured in RPMI-1640 (Gibco, Shanghai, China) supplemented with 10% fetal bovine serum (Gibco) and 100 units/mL each of penicillin and streptomycin (Gibco). L-Asparaginase (L-Asp), prednisone, vincristine, daunorubicin, and chloroquine diphosphate were purchased from Sigma-Aldrich (Shanghai, China), and ruxolitinib (S1378) was purchased from Selleck (Shanghai, China). Primary antibodies against CRLF2 (ab109626) and ATG5 (ab108327) were purchased from Abcam (Shanghai, China); anti-α-tubulin (66031-1) was purchased from Proteintech (Wuhan, China); anti-LC3B (2775S), anti-BECLIN-1 (3738S), anti-GATA3 (5852S), anti-JAK2 (3230S), anti-phospho-JAK2 (Tyr1007/1008) (3771S), STAT3 (12640S), and anti-phospho-STAT3 (Tyr705) (9145S) antibodies were obtained from Cell Signaling Technology (Shanghai, China). Nalm6, REH, U-OCB1, SEM, and 697 B-ALL cell lines were purchased from ATCC (Beijing, China). The details are provided in **Supplementary Table 4.**

**Genome engineering using the CRISPR-Cas9 system**

Single guide RNAs (sgRNAs) were designed using the Zhang laboratory CRISPR design tool (http://crispr.mit.edu)[^3^](#_ENREF_24). sgRNA sequences targeting *GATA3* rs3824662 (**Supplementary Table 5**) were cloned into plasmid pSpCas9(BB)-2A-GFP (PX458, Plasmid# 48138, Addgene) as previously described[^4^](#_ENREF_25). The 100-nt single-stranded oligodeoxynucleotides (ssODNs) repair templates were designed with a homologous genomic flanking sequence centered near the predicted CRISPR/Cas9 cleavage site and containing pathogenic mutations. GM18900 cells were transfected with Cas9- and sgRNA-expressing plasmids and ssODNs via electroporation using Gene Pulser Xcell Electroporation Systems (Bio-Rad, Shanghai, China) according to the manufacturer's instructions. Single colonies were isolated using a FACSAria II flow cytometer (BD Biosciences, Shanghai, China).

**Ectopic *GATA3* expression**

To produce lentivirus overexpressing human *GATA3*, 4×10^6^ 293T cells were cultured in a 10-cm dish for 24 h and then transfected with pFUGW empty vector or *GATA3*. Forty-eight hours after transfection, the virus-containing culture supernatant was harvested and kept in a Lenti-X concentrator (Clontech, Beijing, China). Supernatants were centrifuged at 1,500 × *g* for 45 min at 4 °C. Pellets were resuspended in complete media. SUP B15, Nalm6, 697, REH, SEM, U-OCB1, and Ba/F3 cells (1×10^6^) were transduced with 0.5 mL Lentivirus encoding EV-Blast (empty vector) or 4 μg/mL *GATA3*-Blast with polybrene. Forty-eight hours after transfection, the cells were selected and expanded using blasticidin (6 µg/mL) for 7 days.

**CRISPR-mediated *GATA3* knockout**

CRISPR/Cas9 knockouts were generated following current protocols^5,6^. ALL cells were first transduced with the LentiCas9-Blast vector (Plasmid# 52962, Addgene) and were selected using 6 μg/mL blasticidin (Sigma-Aldrich, Shanghai, China). Next, sgRNAs (**Supplementary Table 5**) targeting *GATA3* were cloned into the LentiGuide-Puro vector (Plasmid# 52963, Addgene) as previously described[^3^](#_ENREF_25). The blasticidin-resistant ALL cells were then transduced with the LentiGuide-Puro vectors containing *GATA3* sgRNAs, and the transduced cells were selected by growing the cells in a combination of 6 μg/mL blasticidin and 2 μg/mL puromycin (Sigma-Aldrich).

***In vitro* cytotoxicity assay**

Cells were seeded in 96-well plates at a density of 30,000 cells per 100 μL per well with either vehicle (DMSO 0.1%) or increasing concentrations of drugs for 72 h. Cell viability was assessed by MTT (Sigma-Aldrich) according to the manufacturer’s instructions. Procedures to determine the effects of certain conditions on cell proliferation were performed in three independent experiments.

**Genotyping**

Germline genomic DNA was extracted from peripheral blood samples obtained during clinical remission (blast % <5% in bone marrow and no blast cells in peripheral blood) in children with ALL. *GATA3* SNPs in the samples were genotyped using Sanger sequencing (**Supplementary Table 5**).

**Quantitative RT-PCR**

Total RNA was extracted by using TRIzol LS reagent (Thermo Fisher Scientific, Shanghai, China) according to the manufacturer’s instructions. Purified total RNA was reverse transcribed to cDNA using PrimeScript™ RT Master Mix (Takara Bio, Beijing, China) according to the manufacturer’s instructions. Gene expression was quantified using the Applied Biosystems QuantStudio 6 Flex RT-PCR system (Thermo Fisher Scientific) and the TB Green™ Premix Ex Taq™ II kit (Takara Bio) according to the manufacturer’s instructions.

**Western blotting**

Cellular protein lysates were prepared using the Cell lysis buffer (Cell Signaling Technology, Shanghai, China) according to the manufacturer’s extraction protocol. Protein quantitation was performed using Pierce™ BCA Protein Assay Kit (Thermo Fisher Scientific). A total of 30 μg of protein was denatured in Laemmli buffer at 95 °C for 10 min and western immunoblotting was performed using TGX 7.5%, 10% and 12% gels (Bio-Rad, Shanghai, China). Protein transfer onto PVDF membranes was performed using the Trans Blot turbo system (Bio-Rad). Immunoblotting was performed using primary antibody mentioned in **Supplementary Table 4**. Secondary anti-rabbit/mouse antibodies were purchased from Cell Signaling Technology. Images were acquired using the ChemiDoc MP Imaging System (Bio-Rad). Image Lab software version 4.1 (Bio-Rad) was used for densitometry analyses of the western blots.

**Cell cycle analysis**

Cells were harvested and fixed in 70% ethanol for 120 min at 4 °C and washed with phosphate-buffered saline supplemented with 1% fetal bovine serum. The cells were treated with 100 µg/mL RNase A (abcam, Shanghai, China) for 1 h at 37 °C in a humidified 5% CO_2_ incubator and stained using 50 µg/mL propidium iodide (abcam) for 30 min at room temperature. The DNA content of the cells was analyzed using FACSCalibur (BD Biosciences, Shanghai, China) and FlowJo v. 10 software (FlowJo, Ashland, OR, USA).

**Luciferase reporter assay**

A 1,120-bp region encompassing rs3824662 was amplified using CloneAmp HiFi PCR Premix (Clontech) and then cloned into the pGL4.23-mini/P vector with a minimal SV40 promoter upstream of the firefly luciferase gene sequence. Proximal promoter regions from *GATA3*, *BECN1,* and *ATG5* were ligated to the pGL4.23-mini/P vector. HEK 293T cells and/or B-ALL cell lines were transfected with pGL4.23 generated constructs. Cells were lysed 24 h after transfection using passive lysis buffer (Promega, E1910). Luciferase activity was measured using a dual-luciferase reporter assay and a Lumat LB9507 luminometer. Experiments were performed in triplicate. As a control for cell number and transfection efficiency, firefly luciferase activity was normalized to that of renilla luciferase. Measurements are presented as a ratio relative to the activity of the pGL4.23-mini/P empty vector.

**Statistical analysis**

All statistical analyses were performed using GraphPad Prism® and/or R (version 3.2.5, https://www.R-project.org); all tests were two-sided. *P* < 0.05 was considered to be statistically significant, *P* < 0.05, *; *P* < 0.01, **; *P* < 0.001, ***; and *P* < 0.0001, ****.

**References**

1. Que LP, Huang K, Fang JP, et al. Reassessment of the risk-stratified GD-2008 ALL protocol. *J Pediatr Hematol Oncol.* 2018;**40**:472-477.
2. Shen S, Chen X, Cai J, et al. Effect of dasatinib vs imatinib in the treatment of pediatric Philadelphia chromosome-positive acute lymphoblastic leukemia: a randomized clinical trial. *JAMA Oncol.* 2020;**6**:358-366.
3. Liu Q, Zhang Y, Li F, Li J, Sun W, Tian C. Upgrading of efficient and scalable CRISPR-Cas-mediated technology for genetic engineering in thermophilic fungus *Myceliophthora thermophila*. *Biotechnol Biofuels.* 2019;**12**:293.
4. Ran FA, Hsu PD, Wright J, Agarwala V, Scott DA, Zhang F. Genome engineering using the CRISPR-Cas9 system. *Nat Protoc.* 2013;**8**:2281-2308.
5. Sanjana NE, Shalem O, Zhang F. Improved vectors and genome-wide libraries for CRISPR screening. *Nat Methods* 2014;**11**:783-784.
6. Shalem O, Sanjana NE, Hartenian E, et al. Genome-scale CRISPR-Cas9 knockout screening in human cells. *Science.* 2014;**343**:84-87.
