# Supplementary material for: Inherited GATA3 variant associated with positive minimal residual disease in childhood B‐cell acute lymphoblastic leukemia via asparaginase resistance
Source: Clin Transl Med. 2021 Aug 23;11(8):e507. doi: 10.1002/ctm2.507 (PMC8382977; doi:10.1002/ctm2.507)
Supplement: Supplementary file 2 — Supporting Information [file CTM2-11-e507-s001.pptx]

## Slide 1
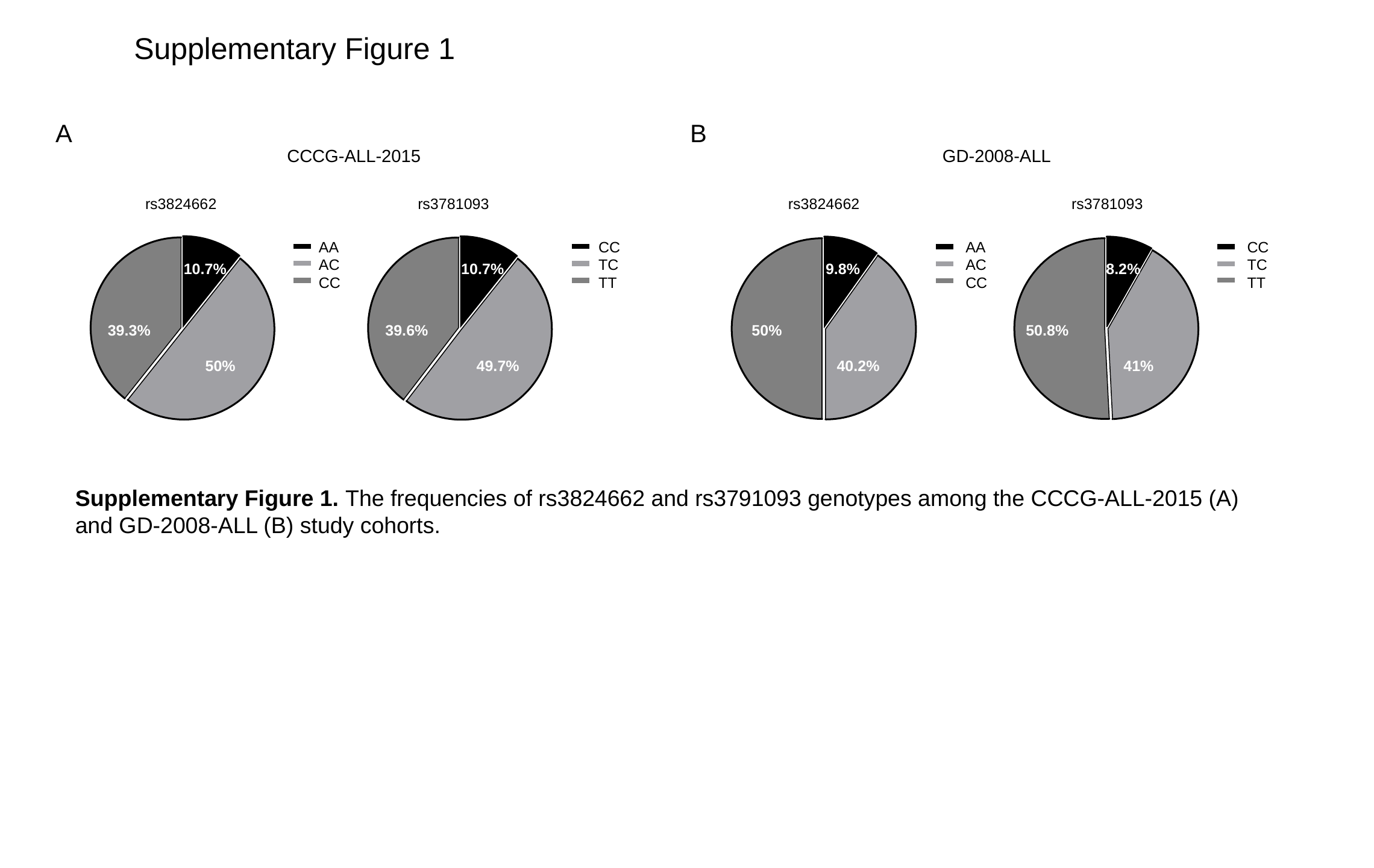

Supplementary Figure 1
A
B
CCCG-ALL-2015
GD-2008-ALL
rs3824662
rs3781093
rs3824662
rs3781093
AA
AC
CC
10.7%
39.3%
50%
CC
TC
TT
10.7%
39.6%
49.7%
AA
AC
CC
9.8%
50%
40.2%
CC
TC
TT
8.2%
50.8%
41%
Supplementary Figure 1. The frequencies of rs3824662 and rs3791093 genotypes among the CCCG-ALL-2015 (A) and GD-2008-ALL (B) study cohorts.

## Slide 2
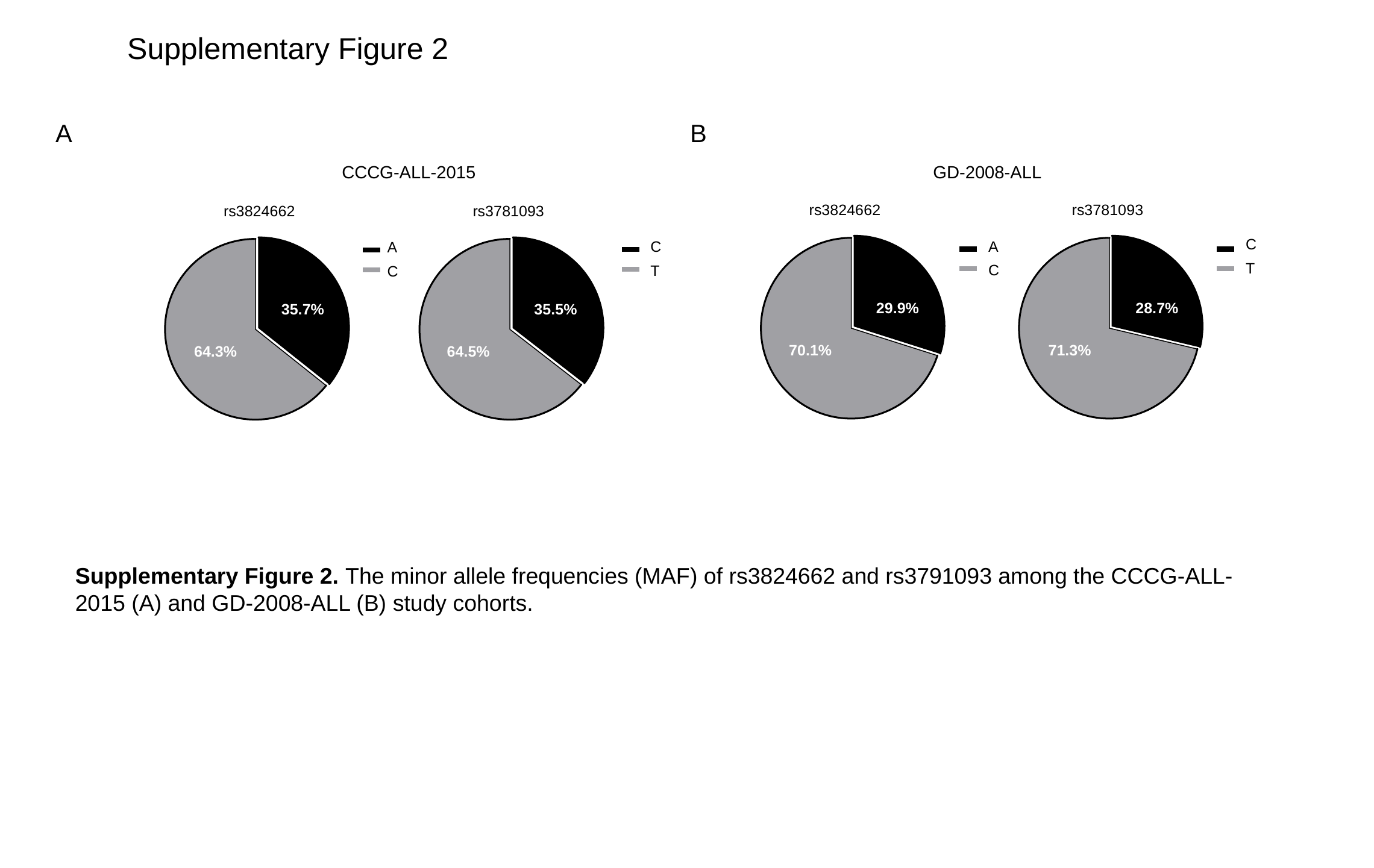

Supplementary Figure 2
A
B
CCCG-ALL-2015
GD-2008-ALL
rs3824662
A
C
29.9%
70.1%
rs3781093
C
T
28.7%
71.3%
rs3824662
A
C
35.7%
64.3%
rs3781093
C
T
35.5%
64.5%
Supplementary Figure 2. The minor allele frequencies (MAF) of rs3824662 and rs3791093 among the CCCG-ALL-2015 (A) and GD-2008-ALL (B) study cohorts.

## Slide 3
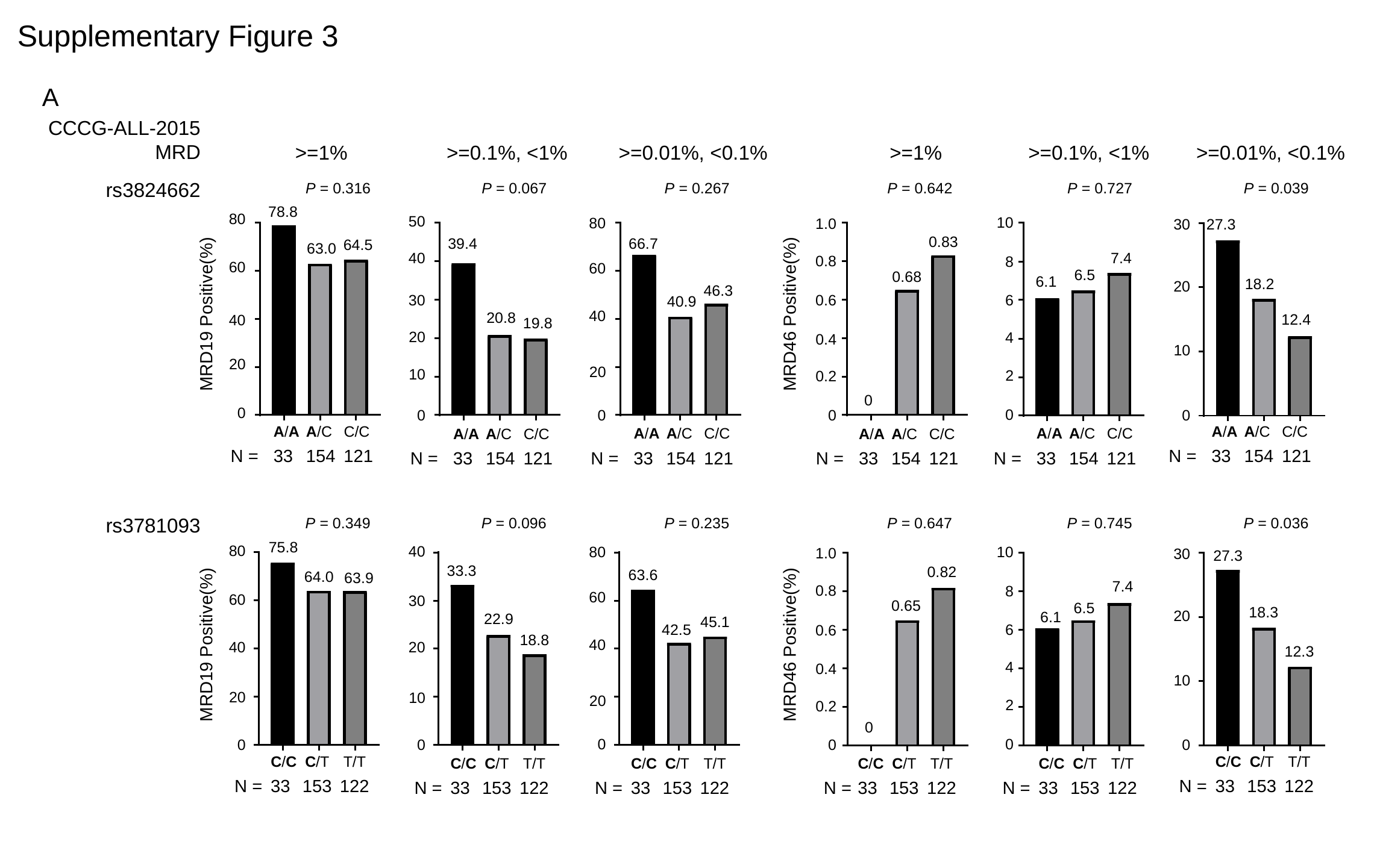

Supplementary Figure 3
A
CCCG-ALL-2015
MRD
>=1%
>=0.1%, <1%
>=0.01%, <0.1%
>=1%
>=0.1%, <1%
>=0.01%, <0.1%
rs3824662
P = 0.316
P = 0.067
P = 0.267
P = 0.642
P = 0.727
P = 0.039
78.8
80
50
10
80
1.0
30
27.3
0.83
66.7
39.4
64.5
63.0
7.4
40
0.8
8
60
60
6.5
0.68
6.1
18.2
20
46.3
30
6
0.6
40.9
MRD19 Positive(%)
MRD46 Positive(%)
40
20.8
12.4
40
19.8
20
4
0.4
10
20
20
10
2
0.2
0
0
0
0
0
0
0
A/A
A/C
C/C
A/A
A/C
C/C
A/A
A/C
C/C
A/A
A/C
C/C
A/A
A/C
C/C
A/A
A/C
C/C
N =
33
154
121
N =
33
154
121
N =
33
154
121
N =
33
154
121
N =
33
154
121
N =
33
154
121
rs3781093
P = 0.349
P = 0.096
P = 0.235
P = 0.647
P = 0.745
P = 0.036
75.8
80
40
80
10
1.0
30
27.3
33.3
0.82
63.6
64.0
63.9
7.4
0.8
8
60
60
30
0.65
6.5
18.3
20
6.1
22.9
45.1
42.5
6
0.6
18.8
MRD19 Positive(%)
MRD46 Positive(%)
40
20
40
12.3
4
0.4
10
20
10
20
2
0.2
0
0
0
0
0
0
0
C/C
C/T
T/T
C/C
C/T
T/T
C/C
C/T
T/T
C/C
C/T
T/T
C/C
C/T
T/T
C/C
C/T
T/T
N =
33
153
122
N =
33
153
122
N =
33
153
122
N =
33
153
122
N =
33
153
122
N =
33
153
122

## Slide 4
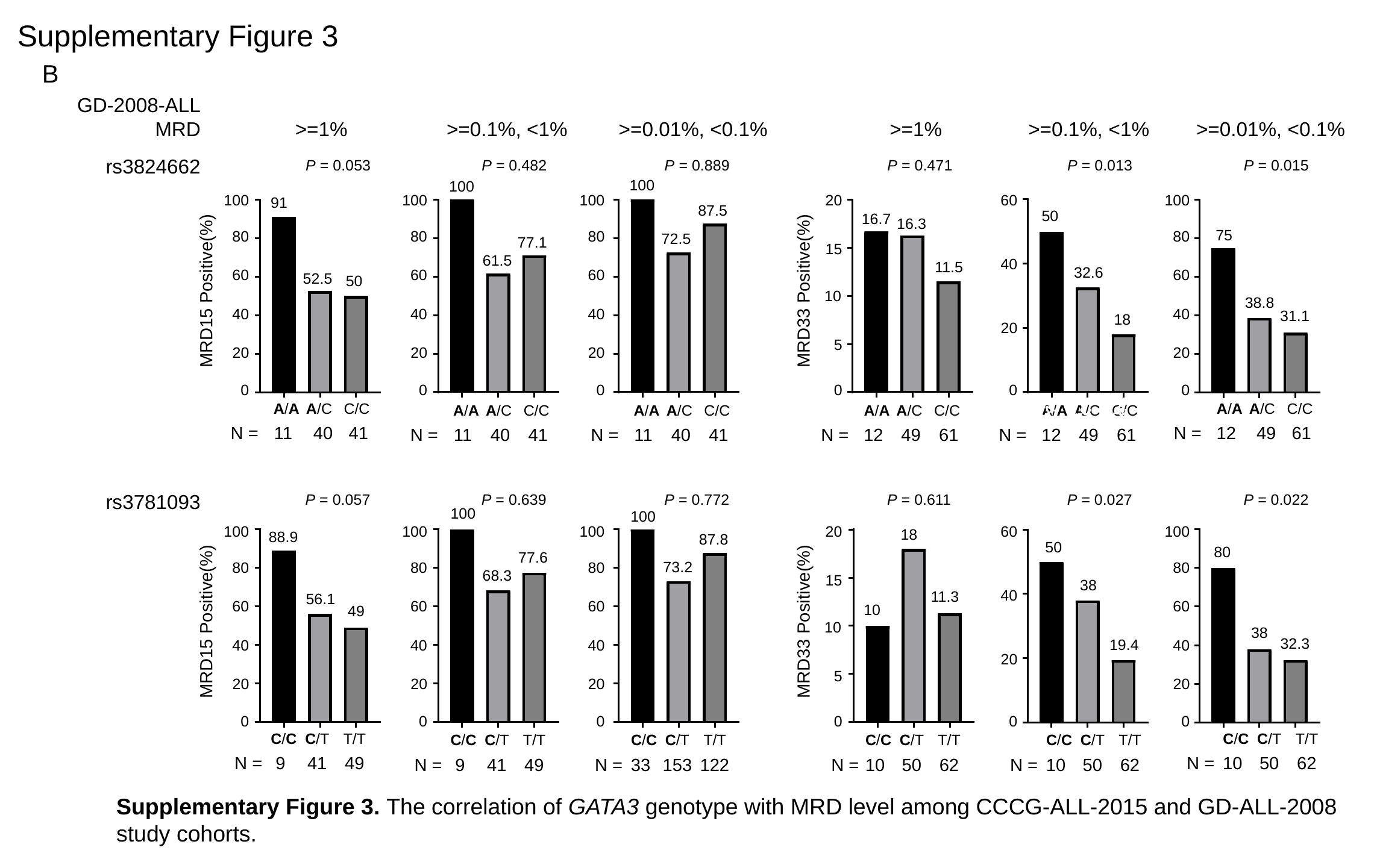

Supplementary Figure 3
B
GD-2008-ALL
MRD
>=1%
>=0.1%, <1%
>=0.01%, <0.1%
>=1%
>=0.1%, <1%
>=0.01%, <0.1%
rs3824662
P = 0.053
P = 0.482
P = 0.889
P = 0.471
P = 0.013
P = 0.015
100
100
100
100
100
20
60
100
91
87.5
50
16.7
16.3
75
80
80
80
80
72.5
77.1
15
61.5
40
11.5
32.6
60
60
60
60
52.5
50
MRD15 Positive(%)
MRD33 Positive(%)
10
38.8
40
40
40
40
31.1
18
20
5
20
20
20
20
0
0
0
0
0
0
A/A
A/C
C/C
A/A
A/C
C/C
A/A
A/C
C/C
A/A
A/C
C/C
A/A
A/C
C/C
A/A
A/C
C/C
N =
11
40
41
N =
12
49
61
N =
11
40
41
N =
12
49
61
N =
11
40
41
N =
12
49
61
rs3781093
P = 0.057
P = 0.639
P = 0.772
P = 0.611
P = 0.027
P = 0.022
100
100
100
100
100
20
60
100
18
88.9
87.8
50
80
77.6
73.2
80
80
80
80
68.3
15
38
40
11.3
56.1
60
60
60
60
10
49
MRD15 Positive(%)
MRD33 Positive(%)
10
38
32.3
19.4
40
40
40
40
20
5
20
20
20
20
0
0
0
0
0
0
C/C
C/T
T/T
C/C
C/T
T/T
C/C
C/T
T/T
C/C
C/T
T/T
C/C
C/T
T/T
C/C
C/T
T/T
N =
9
41
49
N =
10
50
62
N =
33
153
122
N =
10
50
62
N =
9
41
49
N =
10
50
62
Supplementary Figure 3. The correlation of GATA3 genotype with MRD level among CCCG-ALL-2015 and GD-ALL-2008 study cohorts.

## Slide 5
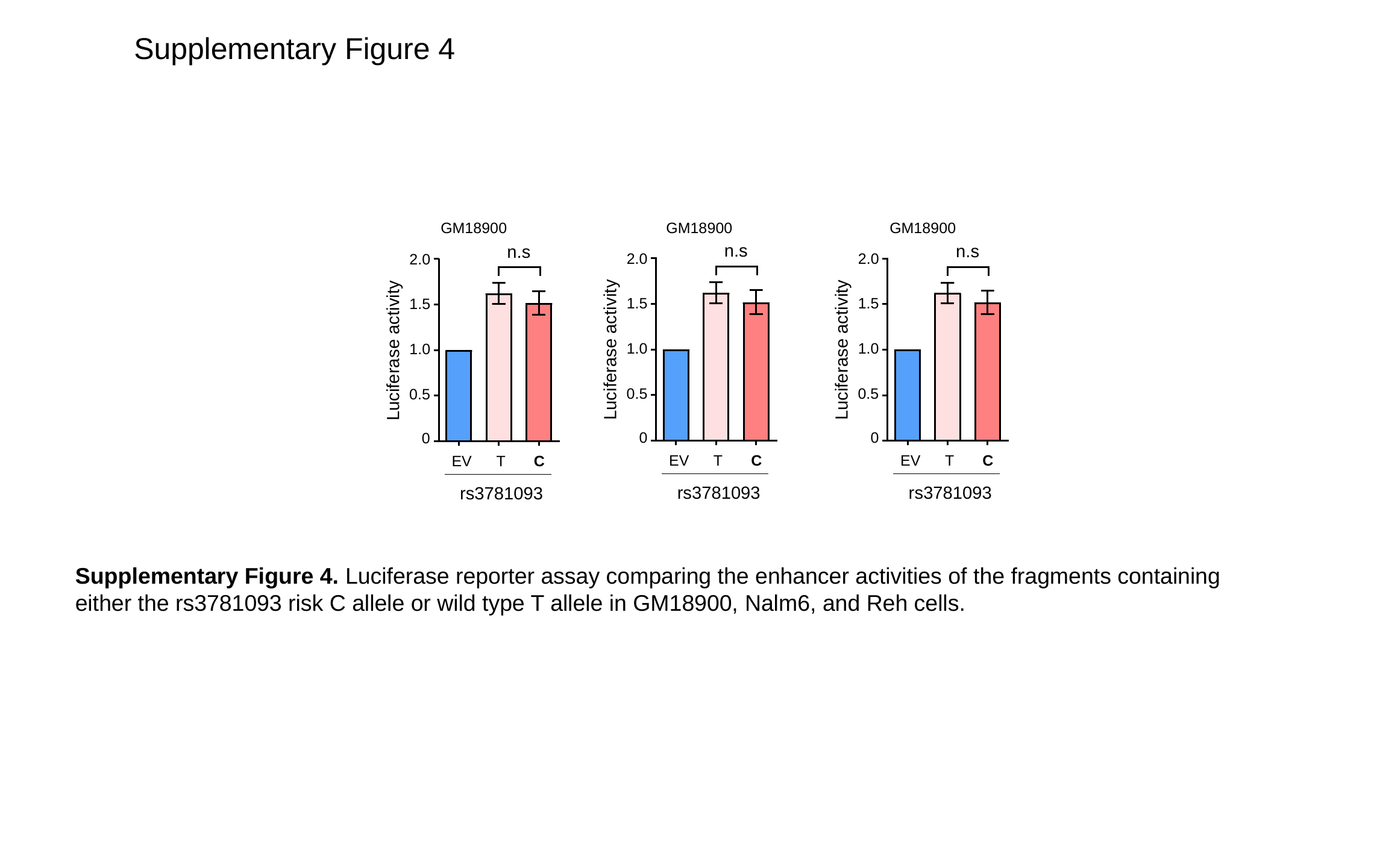

Supplementary Figure 4
GM18900
GM18900
GM18900
n.s
n.s
n.s
2.0
2.0
2.0
1.5
1.5
1.5
1.0
1.0
Luciferase activity
Luciferase activity
1.0
Luciferase activity
0.5
0.5
0.5
0
0
0
EV
T
C
rs3781093
EV
T
C
rs3781093
EV
T
C
rs3781093
Supplementary Figure 4. Luciferase reporter assay comparing the enhancer activities of the fragments containing either the rs3781093 risk C allele or wild type T allele in GM18900, Nalm6, and Reh cells.

## Slide 6
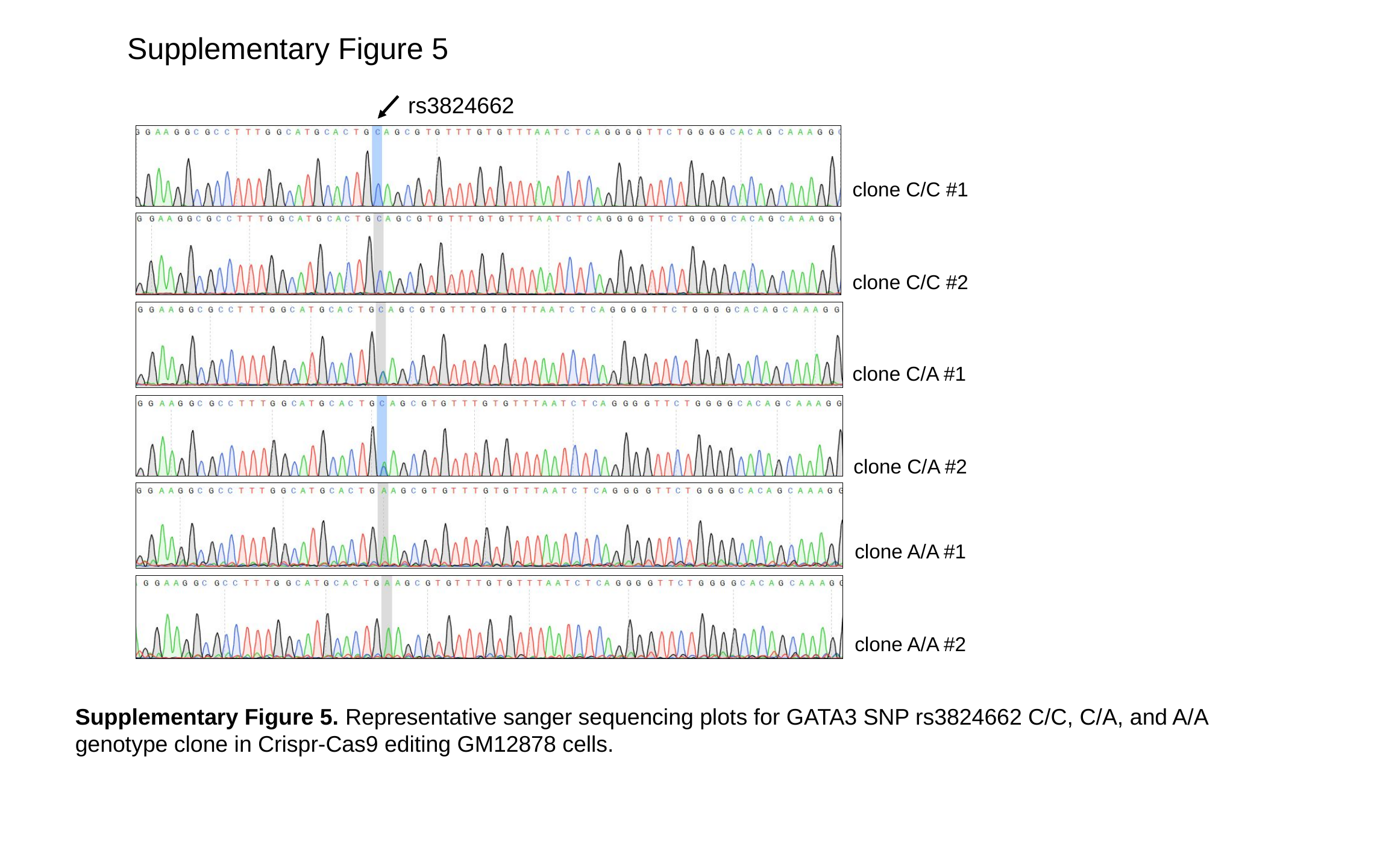

Supplementary Figure 5
rs3824662
clone C/C #1
clone C/C #2
clone C/A #1
clone C/A #2
clone A/A #1
clone A/A #2
Supplementary Figure 5. Representative sanger sequencing plots for GATA3 SNP rs3824662 C/C, C/A, and A/A genotype clone in Crispr-Cas9 editing GM12878 cells.

## Slide 7
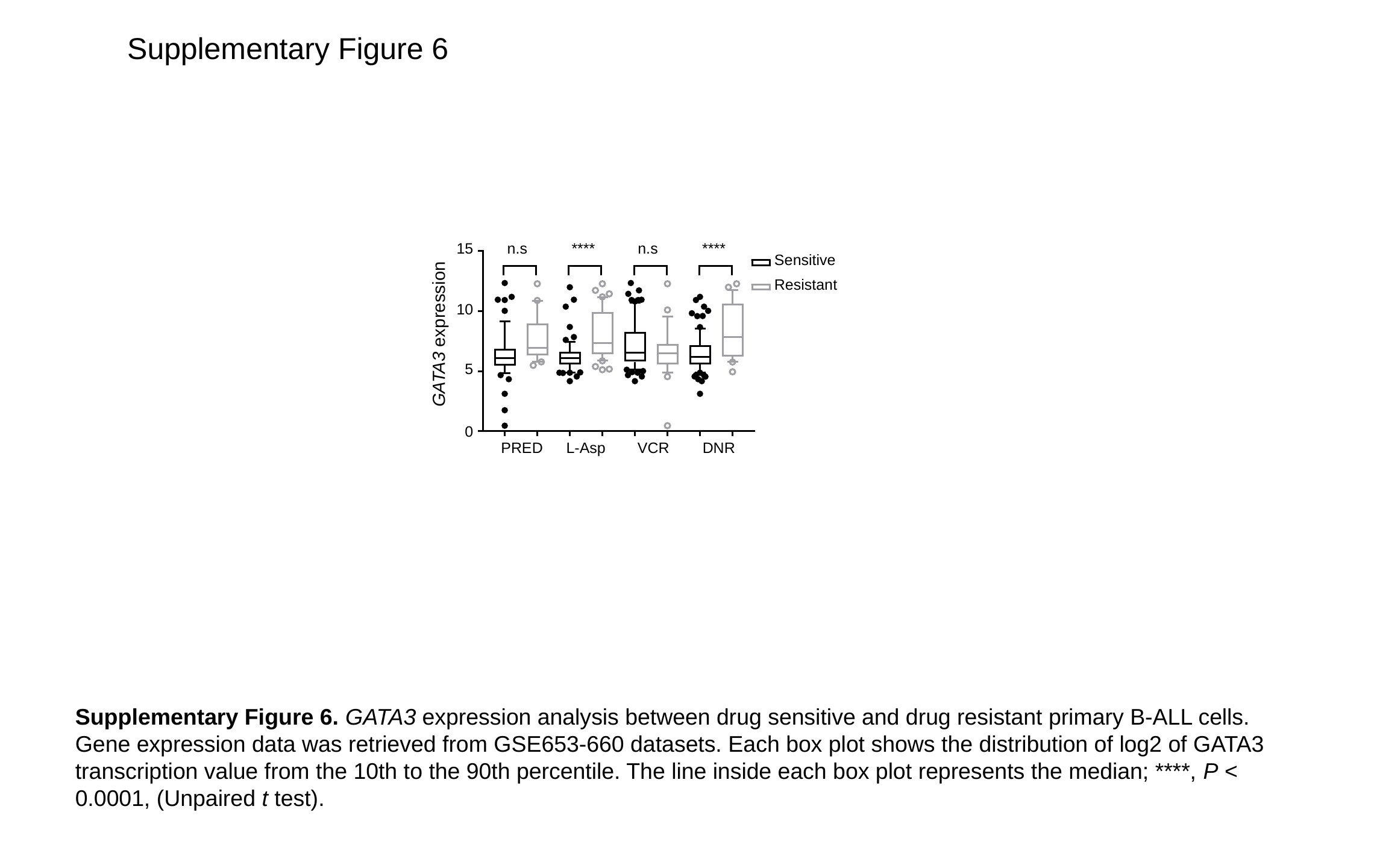

Supplementary Figure 6
n.s
****
n.s
****
15
Sensitive
Resistant
10
GATA3 expression
5
0
PRED
L-Asp
VCR
DNR
Supplementary Figure 6. GATA3 expression analysis between drug sensitive and drug resistant primary B-ALL cells. Gene expression data was retrieved from GSE653-660 datasets. Each box plot shows the distribution of log2 of GATA3 transcription value from the 10th to the 90th percentile. The line inside each box plot represents the median; ****, P < 0.0001, (Unpaired t test).

## Slide 8
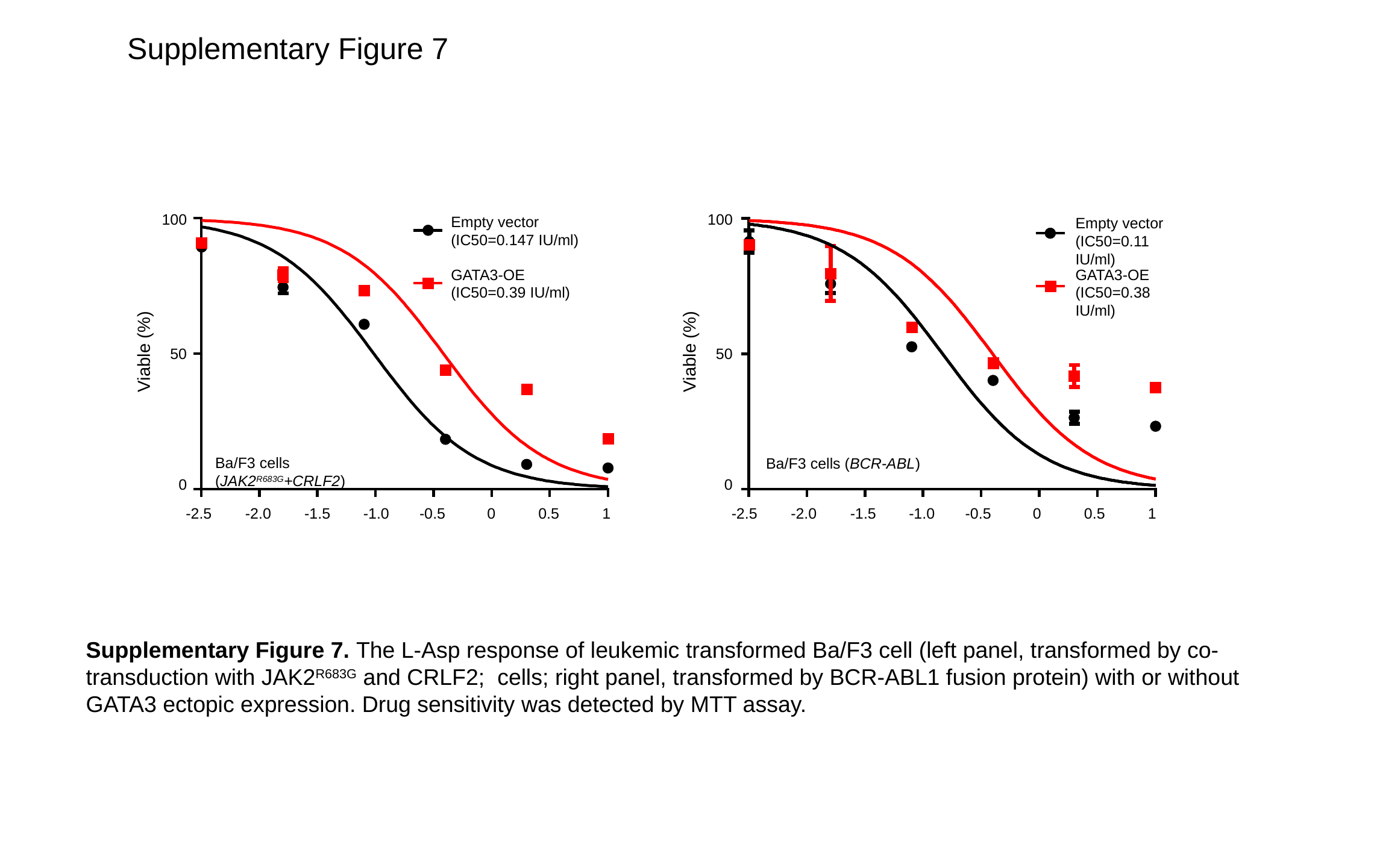

Supplementary Figure 7
100
50
0
Viable (%)
-2.5
-2.0
-1.5
-1.0
-0.5
0
0.5
1
100
50
0
Viable (%)
-2.5
-2.0
-1.5
-1.0
-0.5
0
0.5
1
Empty vector
(IC50=0.147 IU/ml)
Empty vector
(IC50=0.11 IU/ml)
GATA3-OE
(IC50=0.38 IU/ml)
GATA3-OE
(IC50=0.39 IU/ml)
Ba/F3 cells (JAK2R683G+CRLF2)
Ba/F3 cells (BCR-ABL)
Supplementary Figure 7. The L-Asp response of leukemic transformed Ba/F3 cell (left panel, transformed by co-transduction with JAK2R683G and CRLF2; cells; right panel, transformed by BCR-ABL1 fusion protein) with or without GATA3 ectopic expression. Drug sensitivity was detected by MTT assay.

## Slide 9
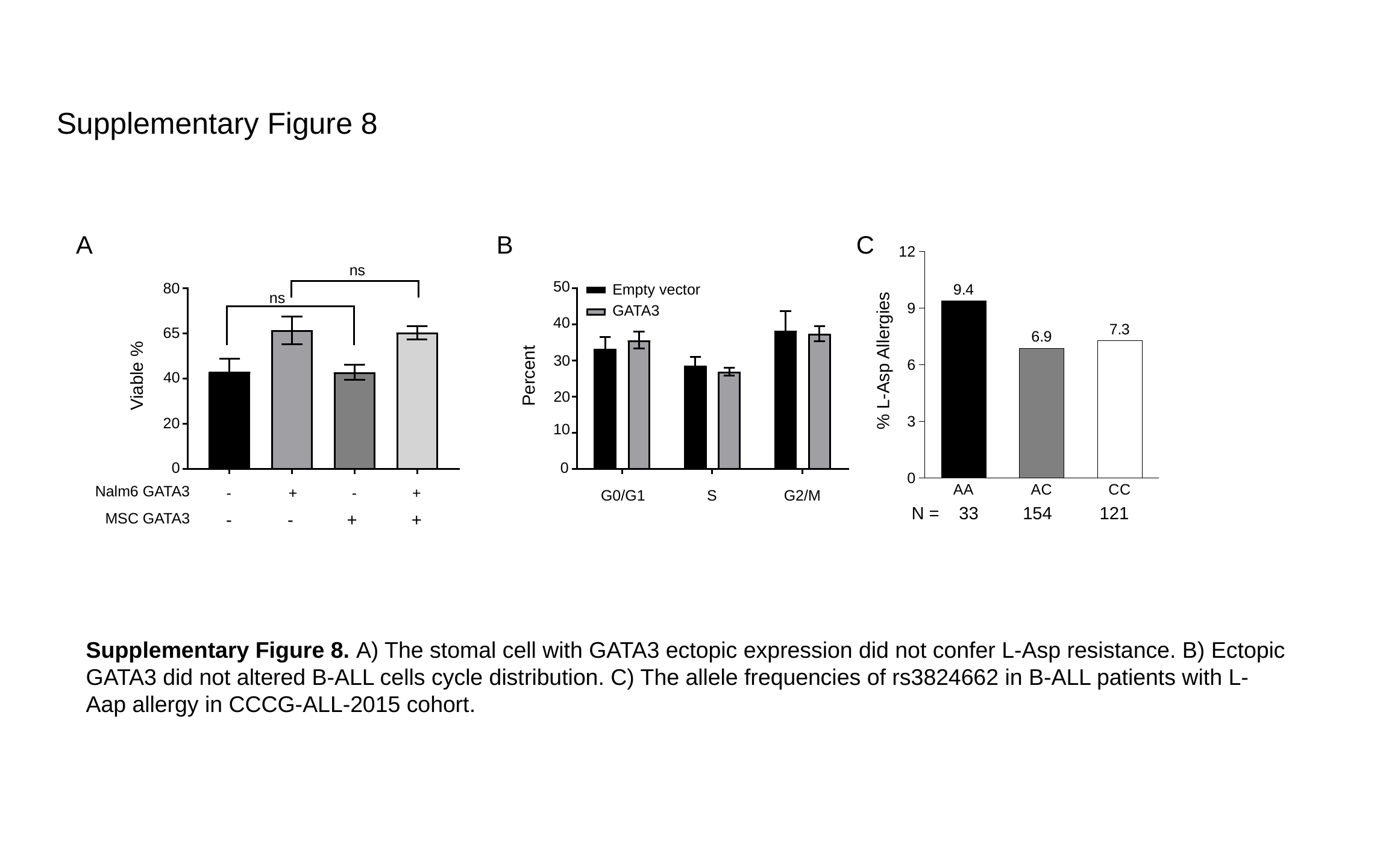

Supplementary Figure 8
A
B
C
### Chart
| Category | |
|---|---|
| AA | 9.375000000000027 |
| AC | 6.87022900763361 |
| CC | 7.27272727272727 |ns
80
ns
65
Viable %
40
20
0
Nalm6 GATA3
-
+
-
+
MSC GATA3
-
-
+
+
50
Empty vector
GATA3
40
30
Percent
20
10
0
G0/G1
S
G2/M
% L-Asp Allergies
N =
33
154
121
Supplementary Figure 8. A) The stomal cell with GATA3 ectopic expression did not confer L-Asp resistance. B) Ectopic GATA3 did not altered B-ALL cells cycle distribution. C) The allele frequencies of rs3824662 in B-ALL patients with L-Aap allergy in CCCG-ALL-2015 cohort.

## Slide 10
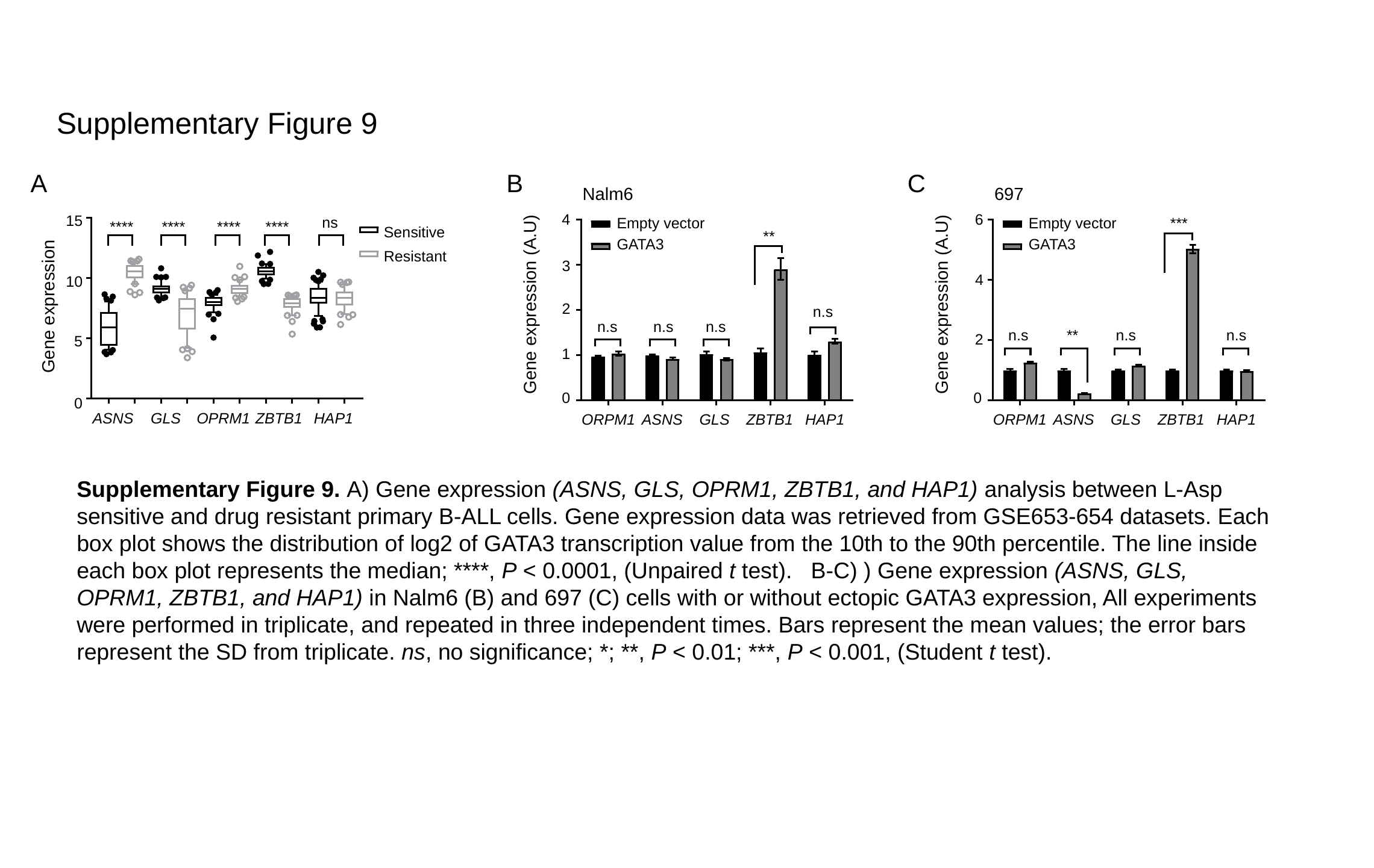

Supplementary Figure 9
A
B
C
Nalm6
697
4
6
15
ns
Empty vector
Empty vector
***
****
****
****
****
Sensitive
**
GATA3
GATA3
Resistant
3
4
10
Gene expression (A.U)
Gene expression (A.U)
Gene expression
2
n.s
n.s
n.s
n.s
n.s
n.s
**
n.s
2
5
1
0
0
0
ASNS
GLS
OPRM1
ZBTB1
HAP1
ORPM1
ASNS
GLS
ZBTB1
HAP1
ORPM1
ASNS
GLS
ZBTB1
HAP1
Supplementary Figure 9. A) Gene expression (ASNS, GLS, OPRM1, ZBTB1, and HAP1) analysis between L-Asp sensitive and drug resistant primary B-ALL cells. Gene expression data was retrieved from GSE653-654 datasets. Each box plot shows the distribution of log2 of GATA3 transcription value from the 10th to the 90th percentile. The line inside each box plot represents the median; ****, P < 0.0001, (Unpaired t test). B-C) ) Gene expression (ASNS, GLS, OPRM1, ZBTB1, and HAP1) in Nalm6 (B) and 697 (C) cells with or without ectopic GATA3 expression, All experiments were performed in triplicate, and repeated in three independent times. Bars represent the mean values; the error bars represent the SD from triplicate. ns, no significance; *; **, P < 0.01; ***, P < 0.001, (Student t test).

## Slide 11
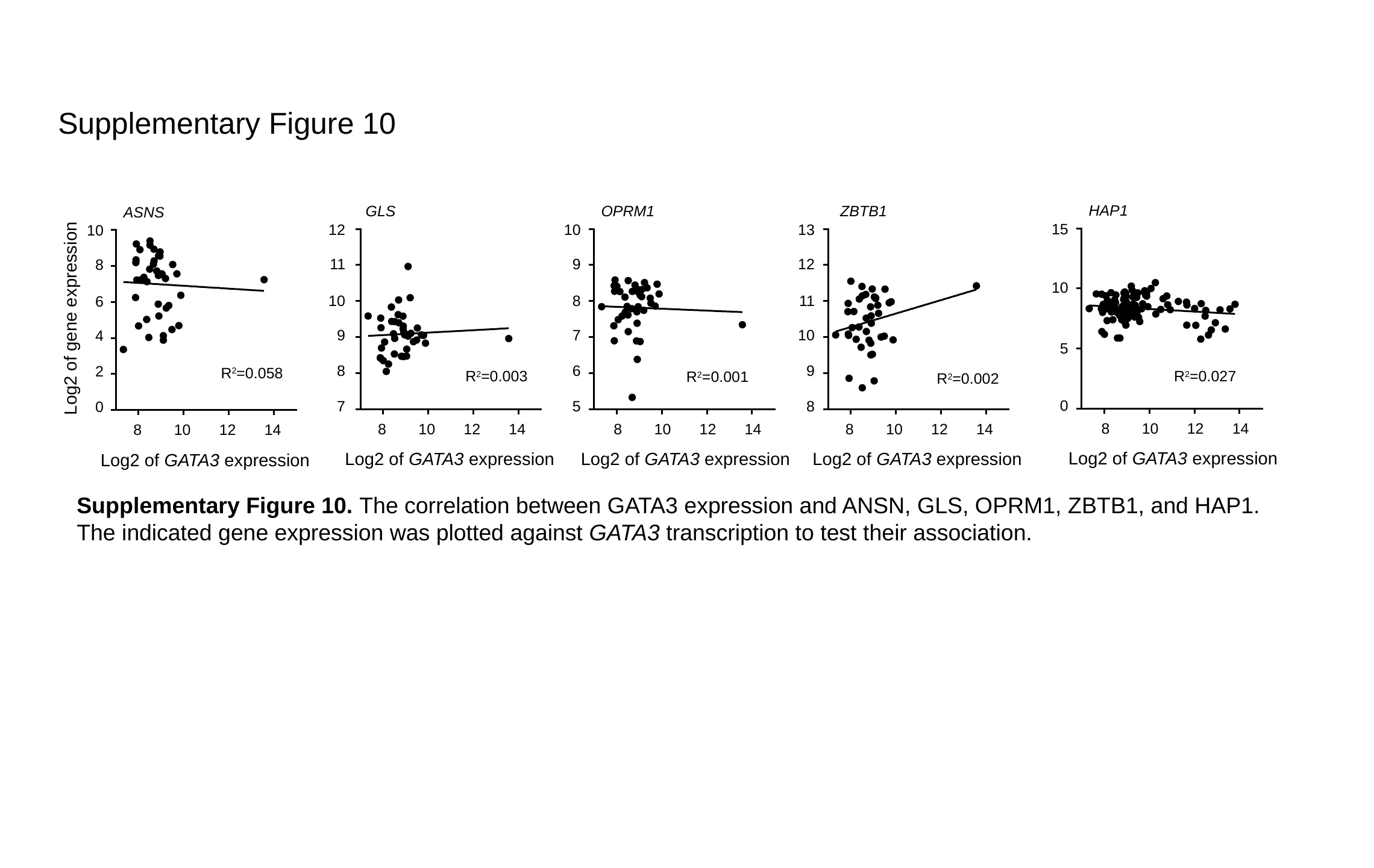

Supplementary Figure 10
HAP1
15
10
5
0
R2=0.027
8
10
12
14
Log2 of GATA3 expression
GLS
12
11
10
9
8
7
R2=0.003
8
10
12
14
Log2 of GATA3 expression
OPRM1
10
9
8
7
6
5
R2=0.001
8
10
12
14
Log2 of GATA3 expression
ZBTB1
13
12
11
10
9
8
R2=0.002
8
10
12
14
Log2 of GATA3 expression
ASNS
10
8
6
4
2
0
Log2 of gene expression
R2=0.058
8
10
12
14
Log2 of GATA3 expression
Supplementary Figure 10. The correlation between GATA3 expression and ANSN, GLS, OPRM1, ZBTB1, and HAP1. The indicated gene expression was plotted against GATA3 transcription to test their association.
